# BRCA1 and Its Vulnerable C-Terminal BRCT Domain: Structure, Function, Genetic Mutations and Links to Diagnosis and Treatment of Breast and Ovarian Cancer

**DOI:** 10.3390/ph17030333

**Published:** 2024-03-04

**Authors:** Tala Ismail, Safa Alzneika, Emna Riguene, Salwa Al-maraghi, Aya Alabdulrazzak, Noof Al-Khal, Sara Fetais, Angelos Thanassoulas, Halema AlFarsi, Michail Nomikos

**Affiliations:** College of Medicine, QU Health, Qatar University, Doha P.O. Box 2713, Qatar

**Keywords:** BRCA1 gene, breast cancer, ovarian cancer, BRCT domain

## Abstract

The *BRCA1* is a tumor suppressor gene that encodes for the BRCA1 protein, which plays a vital role in DNA repair, cell cycle regulation, and the maintenance of genomic stability. The BRCA1 protein interacts with a variety of other proteins that play essential roles in gene regulation and embryonic development. It is a large protein composed of multiple domains. The C-terminal region of the BRCA1 protein consists of two BRCT domains connected by a short linker. The BRCT domains are crucial in protein–protein interactions as well as in DNA damage response and cell cycle regulation through their phosphoprotein binding modules that recognize the phosphorylated protein sequence motif of other kinases. Mutations within the BRCT domain can disrupt the normal function of BRCA1 and lead to an increased risk of developing breast and ovarian cancer. Herein, we explore the structural characteristics of BRCA1, focusing on the BRCT domain, its interactions with key cellular components, and its involvement in various cellular processes. In addition, the impact of BRCT domain mutations on breast and ovarian cancer susceptibility, prognosis, and treatment options is discussed. By providing a comprehensive understanding of the BRCT domain of BRCA1, this review aims to shed light on the role of this important domain in the pathogenesis and potential therapeutic approaches for breast and ovarian cancer.

## 1. Introduction

The *BRCA1* gene, also known as BReast CAncer gene 1, is a tumor suppressor gene. It encodes the BRCA1 protein, which is involved in DNA repair [1]. The DNA repair process is crucial for restoring DNA integrity after spontaneous damage caused by factors such as ionizing radiation, UV radiation, and various chemical agents [2]. The BRCA1 protein interacts with numerous other proteins that play essential roles in gene regulation and embryonic development [2]. BRCA1 is also involved in regulating the cell cycle during cell division and ensuring genome integrity.

BRCA1 works together with another protein called BRCA2, encoded by the *BRCA2* gene, to maintain the genome. Despite their cooperation, BRCA1 and BRCA2 play different roles in DNA damage response and repair processes. BRCA1 is versatile and participates in both checkpoint activation and DNA repair, while BRCA2 acts as a mediator in homologous recombination. While the precise connections between these two proteins are currently unknown, they must exist to compensate for the striking similarity in cancer susceptibility associated with germline mutations in these genes [3].

Both BRCA1 and BRCA2 are involved in maintaining genomic integrity through DNA repair by homologous recombination. Loss of BRCA function leads to genomic instability, which promotes oncogenic transformation of non-tumorigenic cells into tumor-initiating cells and further tumor development [4]. These proteins have been shown to be involved in transcriptional regulation to protect genome stability from oxidative stress caused by reactive oxygen species [4].

BRCA1 is a large multifunctional protein (1863 amino acids in size) composed of diverse domains essential for its important role in maintaining genomic stability and tumor suppression [5]. Mutations in certain domains can potentially affect the stability of BRCA1 and/or its interactions with other protein targets. Such mutations can disrupt the normal function of the BRCA1 protein, increasing the risk of hereditary breast and ovarian cancer [6]. Understanding the structure and function of BRCA1 is crucial for developing targeted therapies and improving cancer risk assessment [6,7]. The C-terminal domain of BRCA1, known as the BRCT domain, plays a critical role in protein–protein interactions as well as in DNA damage response and cell cycle regulation. It has a phosphoprotein binding module to recognize the phosphorylated protein sequence motif pSXXF of other kinases, ATM/ATR kinases (two kinases induced by DNA damage) [6].

Mutations in the *BRCA1* gene are a marker for predicting susceptibility to high-risk cancers, particularly hereditary breast and ovarian cancer syndrome (HBOC), fallopian tube, prostate, and colon cancers. BRCA mutation status is often associated with more severe disease and shorter overall survival [8]. Several studies have attempted to predict the cancer risks associated with unclassified BRCA1 missense variants by performing bioinformatics analyses based on multiple sequence alignment data and protein structure predictions as well as clinical and family history data for some of these mutations. These results suggest that the BRCT domains of BRCA1 are the most highly conserved regions of the protein, which are responsible for the vast majority of cancer-related mutations [9].

## 2. Structure and Function of BRCA1-BRCT Domain

**Figure 1:**
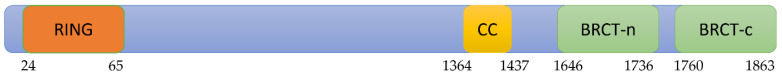
Domain organization of the BRCA1 protein. Human BRCA1 contains an N-terminal RING domain (24–65aa), coiled coil domain (1364–1437aa) and two C-terminal BRCT repeats (1646–1863aa).

The *BRCA1* gene is located on chromosome 17 at 17q21 (from position 43,044,294 bp to 43,125,482 bp) and encodes a 1863 amino acid protein composed of multiple domains, including the N-terminal RING domain (residue 24 to 65), a central region, the coiled coil CC domain (1364–1437), and the C-terminal BRCT domains (Figure 1).

The RING domain is characterized by a conserved pattern that contains seven cysteine and one histidine residue, thus forming two distinct Zn^2+^ binding sites. This configuration leads to the formation of a stable heterodimer complex with the RING domain of BARD1, a protein associated with inhibition of 3′ end processing of mRNA precursors [10].

The N-terminal RING domain coordinates two zinc cations in a cross-braced configuration. This domain plays a central role in E3 ubiquitin ligase activity and promotes the ubiquitination of target proteins, including BRCA1 itself, leading to their modification or degradation. The RING domain is crucial for both DNA repair and the cell cycle checkpoint activity of BRCA1 [6]. The CC domain is located toward the C-terminal domain of BRCA1. This domain is a highly conserved region that is essential for the functional synergy between the activation domains of BRCA1. It contains the binding sites for other proteins and is essential for the function of BRCA1 in DNA damage response and homologous recombination repair [11,12]. The C-terminal region of the BRCA1 protein consists of two BRCT domains connected by a 22-amino-acid linker. Each BRCT repeat consists of three α-helix structures arranged around a four-stranded β-sheet [13]. The interaction between the two BRCT repeats occurs in a head-to-tail structure, mainly involving the α2 helix of BRCT1 and the α1 and α3 helices of BRCT2, with a significant contribution from hydrophobic amino acid residues [14].

**Figure 2:**
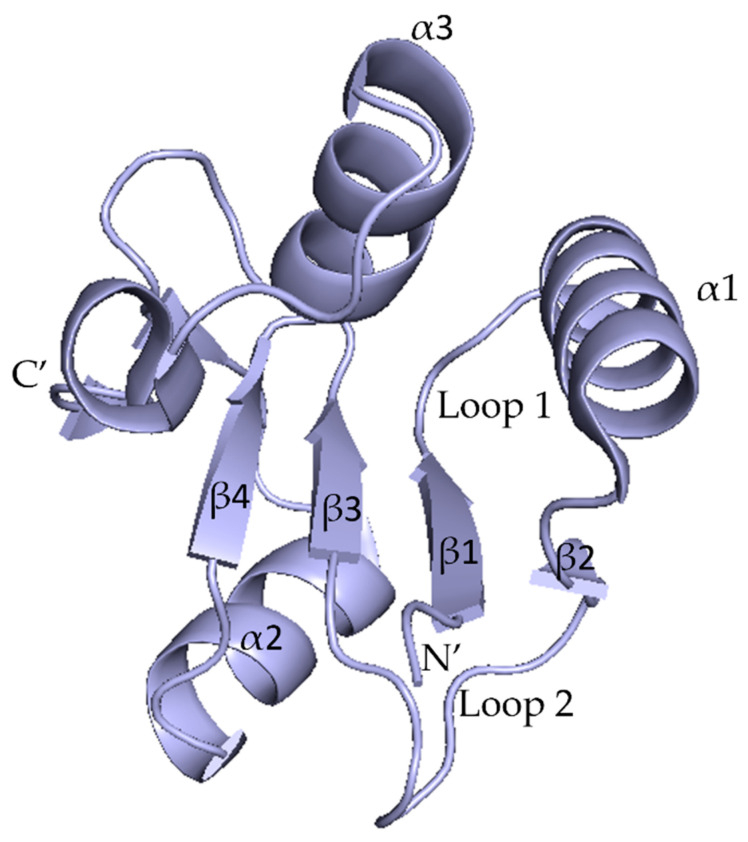
Typical BRCT domain structure, showing the N′-end (head) and C′-end (tail) arrangement, the characteristic (α1, α2, α3) and (β1, β2, β3, β4) motifs, as well as the connecting Loops 1 and 2 (PDB ID: 1CDZ).

The first human BRCT domain structure was resolved by X-ray crystallography using the crystal structure of the N-terminal BRCT from the XRCC1 protein (PDB: 1CDZ). A typical BRCT domain has 90 to 100 amino acid residues and folds as a globular domain with secondary structural elements arranged as βαββαβα [13]. It has been described as a four-stranded parallel β-sheet (β1, β2, β3, and β4) surrounded by two alpha helices (α1 and α3) located at the C-terminal end, a single α-helix (α2) at the N-terminal end, and two surface loops connecting β1 to α1 and α2 to β3 (the overall structure is β1-α1-β2-β3-α2-β4-α3) (Figure 2) [14,15].

Some hydrophobic interactions occur between certain residues of the α1 helix and those of the β1 and β2 sheets. Similarly, other residues in the α2 helix interact with those in the β4 sheet. The highly conserved residues are located in the central β sheets (β1, β3, β4) and the α1 and α3 helices, whereas the α2 helix shows the greatest variability within the BRCT protein family [15]. In addition, the β1, α1, α1–β2 loop, β3, α3, and C-terminal end regions contain five conserved hydrophobic motifs, named A, B, C, D, and E, respectively. However, the alignment of the BRCT sequences shows a low identity. A specific pattern, called the “consensus sequence”, is required for the recognition of other proteins by the BRCA1 BRCT domain. This pattern depends on the size and subsequent rigidity of the hydrophobic core of the interface between the two BRCT repeats. The consensus sequence pSer-X-X-Phe helps BRCA1 identify specific phosphorylated partner proteins. These proteins, such as the DNA helicase BACH1, the nuclease CtIP, and a signaling protein called Abraxas, transmit signals from damaged DNA to downstream targets, such as DNA repair proteins and factors involved in cell cycle regulation [9]. Structural studies have shown that recognition involves a conserved phosphoserine recognition pocket in the NH_2_-terminal BRCT repeat, which is formed by Ser1655, Gly1656, and Lys1702. These components help with identifying the phosphate group [16,17]. For example, the interaction between BRCT domains and other proteins, such as the BACH1 helicase, is mediated by the formation of hydrogen bonds between the pSer residue of the BACH1 protein with Ser1655 (S1655), Lys 1702 (K1702) and the backbone amino group of Gly1656 (G1656). A predominantly hydrophobic groove between the two parts of the target peptide highlights the phenylalanine in the third position. Many BRCT domains in various proteins linked to DNA damage signals are likely capable of recognizing phosphopeptides of specific sequences [9].

The BRCA1-BRCT domains function as modules that bind to phosphoproteins, specifically recognizing the phosphorylated protein-sequence motif pSXXF of various kinases. These BRCA-BRCT domains, classified as class I, interact with the phosphorylated binding partners BACH1, CtIP, and CCDC98/abraxas by recognizing the pSer-X-X-Phe sequence. The neighboring amino acids to this binding motif also influence the binding affinity [13]. In conjunction with a sequence-specific DNA binding module, the BRCT repeat region can function as a transcriptional activation domain. This activity contributes to the ability of BRCA1 to regulate gene expression, including p21 and GADD45, and to modulate the activity of transcription factors such as p53 and ER [9,18]. Furthermore, the tandem BRCT repeats of MDC1 specifically bind to the phosphorylated form of the histone variant H2AX, which is a critical chromatin mark that triggers DNA double-strand break signaling [19]. The accumulation of BRCA1 at DNA damage sites is crucial for its role in DNA damage response and repair. The BRCT domains also play a role in the formation of double-stranded rRNA and the prevention of ribosomal R-loops, thereby contributing to normal cellular functions [19].

The BRCT domain is involved in genomic stability through its involvement in the DNA damage response, especially in the early stages [7,20]. The phosphopeptide binding activity of BRCT domains requires the presence of two BRCT domains in tandem, with the N-terminal stabilizing phosphoserine and the tandem BRCT forming a hydrophobic pocket that stabilizes the aromatic residue at the +3 position of the phospho-ligand [13]. The BRCA1–Abraxas complex plays a crucial role in recruiting BRCA1 to double-stranded DNA breaks, regulating DNA damage, and promoting efficient repair. The numerous cancer-associated BRCT mutations identified in patients alter their ability to bind phosphorylated peptides, highlighting the critical role of BRCT domains in maintaining genome integrity and facilitating essential protein–protein interactions for DNA repair and genomic stability maintenance [21].

## 3. Association of BRCA1-BRCT Domains Mutations and Breast Cancer

Breast cancer is classified into luminal A, luminal B, HER2 overexpression, and triple-negative breast cancer (TNBC), each of which is characterized by different histological and pathological features [21]. In TNBC, which accounts for 15% of all invasive cases, expression of the estrogen and progesterone receptors as well as HER2 is absent. This subtype is more common in premenopausal women, African American women, and individuals with deleterious mutations in the *BRCA* (1/2) genes. TNBC development involves many pathways, with the current focus being DNA repair, particularly the BRCA1 and BRCA2 functions. BRCA1 mutations common in RING, BRCT domains, and the middle region increase the risk of various cancers [21].

BRCA1 has over 1600 recorded mutations, including “founder mutations” in certain populations. The exact mechanism that explains how mutations in the BRCT domain increase the risk of breast cancer remains to be investigated. Nonetheless, research suggests that these modifications have a range of effects on BRCA1 and may potentially increase the risk of breast cancer by affecting its binding to proteins such as CtIP, ABRAXAS1, and BACH1-P [22]. BRCT domain mutations may impair the ability of BRCA1 to recognize phospholipids, limiting its role in the DNA damage repair pathway [6]. In addition, BRCA1-BRCT mutations may affect the subcellular localization of BRCA1 and influence its ability to interact with other proteins involved in DNA damage repair and genomic stability [23]. Furthermore, mutations in the BRCT domain can cause structural and functional defects as well as misfolding of the BRCA1 protein, altering its normal function [24]. Notably, *BRCA1* mutations are most common in the RING domain, exons 11-13, and the BRCT domain, suggesting that mutations in these domains may play a role in increasing breast cancer risk. Notably, Ashkenazi Jews may have founder mutations in the RING and BRCT domains [25,26]. *BRCA1* mutations increase the risk of various cancers, including breast, ovarian, male breast, prostate, colon, rectum, pancreatic, and stomach cancers [4].

**Figure 3:**
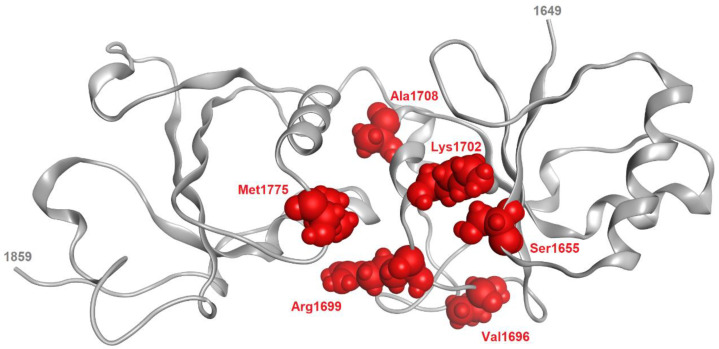
Location of missense mutations associated with severe effects on BRCA1-BRCT structure and function. Source: https://www.nextprot.org/entry/NX_P38398/phenotypes (accessed on 2 December 2023).

Hereditary breast cancer, also known as familial breast cancer (FBC), accounts for 15% of all breast cancer cases, with HBOC syndrome, which is associated with BRCA1 or BRCA2 mutations and significantly increases the risk of breast and ovarian cancer, especially before the age of 50 [27]. In women, the risk of developing a BRCA1 mutation by the age of 70 is 46–87% compared to a 38–84% risk for BRCA2. Destabilizing mutations often alter the primary sequence of tandem BRCT repeats. Frameshift and missense mutations in breast cancer usually result in BRCA1 protein truncation, which is considered functionally deleterious [24]. However, the physiological significance of many such mutations remains unknown due to the lack of BRCT functional assays. Two missense mutations in the BRCT domain were reported in Chinese women with familiar breast cancer, showing similar functions to non-mutated carriers and being associated with reduced growth and stimulated apoptosis [15]. Previous studies identified six BRCT domain missense mutations (Ser 1655, Val 1696, Arg 1699, Lys 1702, Ala 1708, and Met 1775) that contribute to BRCA1 loss of function and disease through protein-destabilizing effects (Table 1) (Figure 3).

A common classification system categorizes BRCA variants as “benign”, “likely benign”, “variant of uncertain significance” (VUS), “likely pathogenic”, and “pathogenic”. While all variants are clinically significant, the clinical implications for VUS are unclear due to insufficient evidence to determine pathogenicity [31]. In the general population, the frequency of BRCA1 or BRCA2 pathogenic variants is predicted to be 1 in 400–500, but it varies by ethnic group and region [32]. In Ashkenazi Jews, for example, the mutation frequency increases to 1 in 40 individuals for certain mutations. Prevalence also varies in other populations, such as African Americans, Latinos and French Canadians, and is influenced by factors such as ethnicity, age at cancer diagnosis, and other demographics [33].

BRCA1/2 mutation carriers have an increased risk of contralateral BC (CBC) with an overall risk of 2.2% up to 2.8% for individuals aged 40 years and younger. In contrast, the probability of developing breast cancer in men with BRCA1 and BRCA2 mutations is 1.2% and 8.9%, respectively [34]. The molecular insight into how BRCA1 mutations can lead to cancer development is still unknown, but the high conservation of BRCT sequences in mammals highlights their functional significance.

## 4. Impact of BRCT Domain Mutations on Breast Cancer Prognosis

Breast cancer is becoming a global health challenge with increasing incidence and mortality rates influenced by changing risk factors, early detection, and improved patient registration [35]. Currently, around 80% of breast cancer patients are 50 years of age and older with survival depending on cancer stage and molecular subtype. Molecular subtyping allows oncologists to categorize breast cancer based on m-RNA-level gene expression, thereby guiding suitable treatments. Genetic factors play a significant role; in particular, 72% of BRCA1 and 69% of BRCA2 mutation carriers have a higher risk of developing breast or ovarian cancers by the age of 80 years. The risk of CBC is higher in mutation carriers, reaching 3% per year and a 10-year risk of 13–40% [36]. According to Kuchenbaecker et al., BRCA1 mutation carriers have a 20-year CBC risk of 40% compared to 26% in non-carriers [34]. Furthermore, women with unilateral breast cancer are at the higher risk of developing bilateral breast cancer (BBC) [35].

In the presence of a BRCA1 mutation, the breast cancer phenotype is likely to be more aggressive, particularly with a TNBC profile, which is found in 10–20% of TNBC cases. Meta-analysis studies show a negative impact of the BRCA1 mutation on overall survival, especially in certain populations, such as Ashkenazi Jewish breast cancer patients [36]. However, there are conflicting results, with the POSH study suggesting higher ovarian cancer for BRCA carriers, possibly due to high sensitivity to chemotherapy [37]. The interplay between BRCA mutations, TNBC, and treatment outcomes highlights the complexity of breast cancer prognosis [38].

Despite negative BRCA1/2 testing in some BBC patients, genetic susceptibility remains unclear. The role of BRCA1 germline mutations in breast cancer prognosis is still debated due to their small proportion and requires further investigation. Oncologists are conducting more detailed studies to determine whether BRCA mutation status can be a reliable prognostic factor, which is crucial due to the approximately 30% increased risk of death for BRCA1 mutation carriers [38]. Furthermore, with its poor prognosis, TNBC has aggressive clinical characteristics, emphasizing the need for accurate prognostic factors [39]. BRCA mutation testing is crucial for susceptible patients and provides a more reliable prognosis. However, additional prospective studies with larger samples, particularly in certain subgroups such as TNBC patients, are needed to refine our understanding of the intricate relationship between genetics and breast cancer prognosis [40].

## 5. Association of BRCA1-BRCT Domain Mutations and Ovarian Cancer

The link between BRCT domain mutations in BRCA1 and susceptibility to ovarian cancer becomes clear when considering the complex relationship between genetic alterations and disease manifestation. Ovarian cancer is characterized by its complexity and diversity and has a significant correlation with specific mutations in the BRCT domain. This underlines the critical interplay between genetic predisposition and susceptibility to the disease [7]. The identification of different BRCA1-associated ovarian cancer subtypes, such as high-grade serous ovarian cancer, further emphasizes the importance of understanding the genetic landscape to develop targeted therapeutic approaches.

The risk of ovarian cancer in BRCA1 mutation carriers, which ranges from 39 to 46% by the age of 70, and for BRCA2 mutation carriers, ranging from 10 to 27% by the same age, underscores the impact of these genetic alterations on disease incidence [1]. The association between BRCT domain mutations and ovarian cancer is particularly relevant to BRCA1’s essential role in maintaining genomic stability and repairing DNA damage [3]. Furthermore, the study by Gorodetska et al. highlights that BRCA1 and BRCA2 mutations are contributing factors to approximately 20% of all ovarian cancers [4]. The high-grade diagnoses of BRCA-related ovarian cancer, exceeding 70%, and the lack of correlation between cancer grade and age at diagnosis highlight the urgency of understanding the impact of BRCA1-BRCT domain mutations in the context of ovarian cancer susceptibility. Furthermore, the involvement of the homologous recombination DNA repair pathway, which is frequently affected by BRCA1 and BRCA2 mutations, further underscores the link between these genetic alterations and the development of ovarian cancer.

Mutations in the *BRCA1* and *BRCA2* genes lead to genetic alterations and cell cycle dysregulation, including the cyclin E1 (*CCNE1*) genes [41]. CCNE1 amplification events, occurring rarely with BRCA inactivation, are crucial in G1/S cell cycle transition. They activate CDK2 and accelerate the cancer cell progression from the G1 to S phase. This enhances genomic instability and disrupts genes involved in cellular survival and proliferation. This amplification was found in 19.1% of ovarian cancers [42]. The *BRCA1* absence or mutation can increase the sensitivity of cells to DNA crosslinkers, such as cisplatin [43].

Ovarian cancer subtyping based on genomic profiling aims to improve survival through appropriate treatments, including immunotherapy. Initially, ovarian cancers were classified into three subtypes: subtype 1 (low immunity), subtype 2 (median immunity), and subtype 3 (high immunity). Further analysis of the Cancer Genome Atlas cohort identified four subtypes, with the C3 subtype having the highest immune signatures, an increased number of tumor-infiltrating immune cells, and a greater expression of immune checkpoints, particularly in patients with the BRCA1 mutation. The C3 subtype has a better survival prognosis compared to other subtypes [44].

Specific parameters, including the tumor microenvironment, immune cells, immune checkpoint molecules, BRCA1/2 mutations, genetic oncology, and prognosis, help identify patients eligible for immunotherapy [45]. The C3 subtype, with its high expression of checkpoint receptors such as CTLA4 ligand CD86, CTLA4, and LAG-3, may be more responsive to checkpoint inhibitors [45]. BRCA1 mutation patients are more common in C3, while BRCA2 mutation patients are mostly ovarian cancer C2-subtype, each having different prognoses. C2 presents the worst survival prognosis, while C3, which has higher immune activity, has a significantly better prognosis [45]. Each subtype has specific cellular pathways. According to Gene Ontology (GO) analysis, the immunoglobulin complex, the circulating immunoglobulin complex, and immunoglobulin receptor binding are significantly promoted in C3. C3 shows increased immune cell invasion, higher anti-tumor immune activity, and a notable expression of *HLA* genes [45]. Ovarian cancer with BRCA1/2 mutations shows higher immune infiltrates, including CD3+ and CD8+ T-cells, high PD-1 expression, and an association with BRCA1 mutation or loss of function, suggesting possible suitability for immune checkpoint blockade therapy [44,46].

## 6. BRCA1-BRCT and Signaling Pathways in Breast and Ovarian Cancer

Cancer, a life-threatening disease resulting from uncontrolled cell proliferation, arises when the tightly regulated cell cycle loses control due to mutations. The cell cycle checkpoint, essential for preventing genetic errors, is compromised in cancer-associated mutations, leading to continuous cell division [47]. *BRCA1*, a cancer susceptibility gene crucial for cell cycle regulation and DNA repair, undergoes mutations that disrupt its structure and contributing to genomic instability, transforming cells into cancer-initiating cells (CSCs) [48]. Clinically significant BRCT mutations or the absence of brca1 lead to cancer development [49]. Functionally, BRCA1 is involved in the Breast Cancer Susceptibility Protein-Associated Genome Surveillance Complex (BASC) and plays a key role in detecting and repairing DNA damage by interacting with proteins such as Rad51 [50].

**Figure 4:**
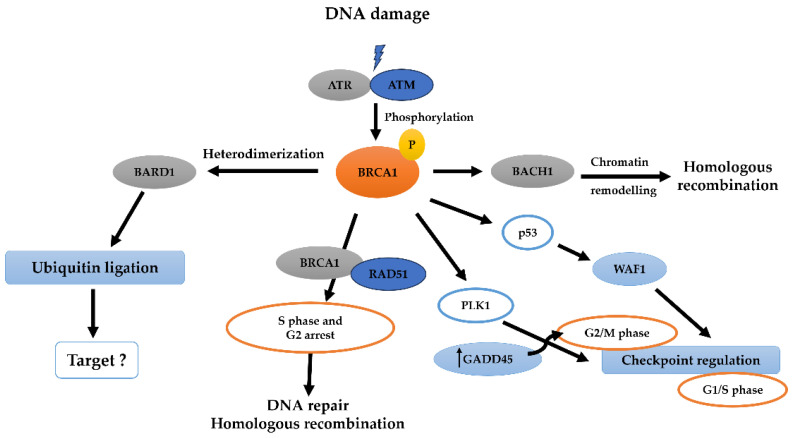
The network associated with BRCA1. The BRCA1 network is a crucial component of pathways governing DNA repair, cell cycle progression, and ubiquitylation. DNA damage, depicted at the top of the figure, is considered a key trigger for BRCA1 activation. Various damage sensors, including ataxia telangiectasia mutated (ATM) and other kinases, become active in response to DNA damage. Downstream targets of BRCA1 activation involve p53. The formation of a complex between BRCA2 and RAD51, interacting with FANCD2 bound to BRCA1, promotes S-phase or G2 arrest. BRCA1, in conjunction with BARD1, forms a heterodimer that activates the ubiquitin–ligase function of BARD1, although its specific targets remain unknown. Homologous recombination for DNA repair is facilitated by the BRCA1-associated surveillance complex. Complexes between BRCA1 and BACH1 mediate chromatin remodeling and homologous recombination. Additionally, BRCA1 interacts with CHK1 and polo-like kinase 1 (PLK1) to regulate the G2/M and G1/S checkpoints, potentially through GADD45, thereby linking BRCA1 to the regulation of apoptosis (modified from [52]).

BRCA1’s regulatory functions extend to the activation of *GADD45* and *P21Waf1/Cip1* genes in response to DNA damage, inducing cell cycle arrest at various phases for repair [51]. The BRCA1-BRCT domains facilitate interactions with CtIP, BACH1 helicase, and AKT, regulating cell cycle phases, the ubiquitination of phosphorylated AKT (pAKT) and inhibiting cell growth by interacting with transcription factors like p53, Myc, STAT1, and CtIP [52]. BRCA1’s mutation causing nuclear pAKT accumulation interferes with nuclear targets like FOXO3a [53]. The role of BRCT interaction with CtIP and its activation of transcription, including interaction with RNA polymerase holoenzyme, remains unclear. BRCA1, frequently mutated in hereditary breast and ovarian cancers (HBOCs), is crucial for maintaining genomic stability, activating DNA damage repair (DDR), mediating ubiquitination, modifying chromatin, and influencing apoptosis and gene regulation (Figure 4). Insufficient BRCA1 (haploinsufficiency) impairs DDR and reduces the ability to repress estrogen receptor (ER-α) signaling, thus inducing genetic mutations and leading to cancer in mammary and ovarian epithelial cells [54].

The tissue-specific patterns of BRCA1-associated cancers emphasize the importance of ovarian hormones, particularly progesterone, in pathogenesis [43]. Progesterone, known for its role in regulating reproductive processes, has been linked to breast cancer, and synthetic progesterone-like compounds increase the risk of cancer. Furthermore, susceptibility to tumorigenesis associated with BRCA1 mutations is observed specifically in women in hormone-related cancers, highlighting tissue-specific interactions with ovarian hormone signaling pathways [43]. Research shows that BRCA1 regulates the progesterone pathway via the progesterone receptor (PR). BRCA can inhibit ER-α signaling through direct interaction and inhibition as well as indirectly by targeting its downstream effectors. Additionally, mutated BRCA1 increases ER-α expression levels in ovarian cancer cells, indicating tissue specificity. ER serves as a substrate for BRCA1 ubiquitin ligase activity, and mutations in ER-α ubiquitination sites may negate BRCA1-mediated ER-α inhibition [54].

Additionally, the role of BRCA1 in suppressing ER-α signaling, especially in ovarian cancer cells, underscores its tissue-specific effect [54]. The potential therapeutic target of progesterone signaling offers a non-surgical prophylactic approach to prevent ovarian and breast cancer in BRCA1 mutation carriers and provides a comprehensive overview of the complex interplay of genetic mutations, DNA repair mechanisms, and hormone signaling pathways in cancer development [55,56].

## 7. Genetic Testing and Screening for BRCA1 Mutations

Numerous genetic variations, affecting over 3% of the population, lead to many diseases, such as breast and ovarian cancers. The identification of cancer-related genes promoted the incorporation of molecular genetics into oncology care and highlighted the global concern of cancer susceptibility. The increased demand for genetic services highlights the importance of predictive testing and advancements in mitigating cancer risks in mutation carriers [57]. The identification of an increased number of founder mutations across diverse populations is anticipated to enhance understanding and accessibility. Risk reduction strategies such as prophylactic oophorectomy [58] and bilateral mastectomy [59] contribute to lowering cancer risks. Given the prevalence of these variants, organizations like ASCO advocate for universal genetic testing, particularly for woman with epithelial ovarian cancer.

To screen for BRCA1/2 mutations, a clinical assessment based on family and medical history is typically performed. The National Comprehensive Cancer Network Clinical Practice Guidelines in Oncology (NCCN Guidelines) includes two primary levels of risk assessment, evaluating extended genetic risk factors followed by clinical characteristics as well as family history of breast cancer for BRCA1/2 mutation carriers [60]. Genetic testing is recommended, particularly for patients with an early cancer diagnosis, such as tubo-ovarian/primary peritoneal high grade serous carcinoma, to enable prevention, early detection, and targeted therapeutic approaches [61]. Identifying BRCA mutation carriers in a family improves prevention, leads to early detection, and enables specific therapeutic approaches and chemoprevention of recurrence. In relation to the population, the detection of founder mutations permit an early, rapid and more cost-effective molecular diagnosis of breast cancer [62]. However, challenges such as low socioeconomic status and lack of awareness contribute to delayed diagnosis [63]. It is recommended that all breast and ovarian cancers patients undergo genetic testing to improve treatment and survival and reduce the risk of disease for asymptomatic family members [63]. For other cancers, such as non-mucinous epithelial ovarian, peritoneal or fallopian tube cancer, germline testing is recommended, especially if a mutation is detected during tumor screening. Germline testing is useful for tumors resulting from pathogenic mutations identified in tumor tissue of germline origin [61].

Furthermore, comprehensive multigene panel testing is now required instead of single gene testing. This approach identifies more *BRCA1* carriers and individuals with mutations in high risk-associated genes and offers advantages in term of cost, turnaround time, and ease of interpretation [45]. Screening guidelines are valuable tools for clinical decision making. They are based on data verification and help provide recommendations for mutation carriers. To date, many developed countries have established some guidelines for breast cancer screening. Genetic screening, such as identifying pathogenic variants, has become a standard practice in many countries to help scientists develop prevention strategies, notably MRI screening and risk-reducing surgical interventions, to reduce cancer-related mortality and morbidity. Characterizing the most common BRCA mutations and their geographical distribution is also helpful to improve screening within a population [64]. The NCCN and the American College of Obstetricians and Gynecologists (ACOG) recommend that genetic testing should be mandatory for patients with a family history of cancer or other criteria (Table 2) [63].

Understanding the geographic distribution of prevalent BRCA mutations supports efficient mutational screening within populations. In addition to genetic testing, prevention programs for breast and ovarian cancers should be established, which include intermediate processes of data analysis and comparison of outcome indicators [64].

## 8. Treatment Strategies for BRCA1 Mutation Carrier Patients

The increasing global prevalence of breast and ovarian cancers highlights the urgent need for efficient and targeted treatments. Developing novel therapeutic approaches for these cancers is crucial to refine prognosis, provide more effective treatments and minimize side effects [44]. Innovative strategies aim to improve the scientific understanding of cancer biology, particularly in early-stage carcinomas, thereby enabling early detection and preventive measures. This contributes to improved long-term survival rates, and it allows cancer to be treated as a chronic disease with fewer hospitalizations and minimal side effects [44]. The standard treatment protocol for breast cancer patients typically includes surgery, radiation, chemotherapy followed by hormone therapy, targeted therapy, and immunotherapy [44].

### 8.1. Surgery

For breast cancer, treatment depends on whether the patient has a *BRCA* gene mutation or not. Previous studies have shown that female BRCA mutation carriers are more susceptible to developing a secondary cancer, whether bilateral or contralateral. This led oncologists to recommend bilateral mastectomy to increase their survival rate by 90% [65]. Patients with BRCA mutations have few treatment options, such as surgery and chemotherapy.

Risk-reducing surgeries have many preventative effects. Hysterectomy and bilateral salpingo-oophorectomy help prevent endometrial and ovarian cancer. They are recommended before the age of 40. To reduce postoperative pain, a laparoscopic approach can be helpful to achieve faster recovery and increase the patient’s quality of life [66]. In a woman with a pathogenic BRCA1/2 variant, surgery is required to remove the predominant site and the potential origin of the cancer, which is the fimbriae or the distal part of the fallopian tube. Both ovaries and fallopian tubes should be removed. Furthermore, global guidelines recommend performing surgical procedures, such as a laparoscopic examination of all peritoneal sites and pelvic washing, to detect hidden metastatic sites [66].

### 8.2. Chemotherapy

Taxanes are microtubule-stabilizing chemotherapeutic agents that are responsible for arresting cell proliferation. Docetaxel and paclitaxel have been the most common chemotherapy drugs used in the treatment of breast cancer since 1993 and 1995, respectively. However, in the subgroup of hormone-negative cancers, *BRCA1* mutation carriers are less sensitive to taxane than non-*BRCA* mutation carriers [67]. An approach used for neoadjuvant chemotherapy, consisting of a combination of anthracycline and taxane, demonstrated a pathological complete response (pCR) in 46% of BRCA mutation carrier patients compared to sporadic cancers, which had 22% pCR. A recent meta-analysis demonstrated that a taxane-based therapy may be a better treatment than anthracycline-taxane for advanced breast cancer patients. Both molecules lead to similar clinical outcomes with lower toxicity for taxane [67]. In hereditary BRCA1-related cancers, patients showed a reduced response to taxane chemotherapy compared to other neoadjuvant chemotherapy with platinum agents such as cisplatin and carboplatin [67]. In human epidermal growth factor receptor 2 (HER2)-negative metastatic breast cancer, the use of PARP inhibitors is recommended with the use of olaparib or talazoparib considered as an alternative option to chemotherapy [68].

### 8.3. Hormone Therapy

Hormone therapy (HT) helps to reduce the risk of breast cancer recurrence and mortality. It is based on the impairment of hormone production or disruption of hormone receptor signaling, which leads to the prevention of tumor growth. This treatment is offered to breast cancer survivors up to ten years after diagnosis. The most commonly used HTs are Selective Estrogen Receptor Modulators (Tamoxifen) and Aromatase Inhibitors (Letrozole, Anastrozole and Exemestane) [69]. The use of HT provides a significant reduction in breast cancer recurrence by 40% and mortality by one-third. Despite its clinical effectiveness in preventing recurrence, many cancer survivors do not take their HT as prescribed. Approximately half of all women take less than 80% of their prescribed dose, and up to 50% discontinue treatment within the first five years. The crucial factor for cancer-free survival is adherence and persistence to HT [69].

### 8.4. Targeted Therapy, like PARPS Inhibitors

Poly (ADP-ribose) polymerases (PARPs) are enzymes that are heavily involved in repairing DNA damage. Their activation is induced by DNA damage. At the site of damage, PARP synthesizes a polymer that is responsible for attracting the DNA repair complexes. The PARP inhibitors (olaparib, nilaparib) target PARP enzymes to kill tumor cells by inhibiting their DNA damage repair mechanism, leading to chromosomal instability followed by cell cycle arrest and apoptosis [68]. They increase DNA single-strand breaks in BRCA1-defective cells. In fact, these inhibitors are used in BRCA-related cancers because they act on tumors with defective genes through a mechanism called “synthetic lethality” and are most commonly used in tumors with homologous recombination dysfunction. Previous clinical trials have shown that the use of PARP inhibitors was beneficial in the treatment of BRCA germline mutation carriers and can be used in non-*BRCA* mutation carrier patients [70]. Patients with advanced TNBC harboring germline mutations in the *BRCA*(1/2) genes show a significant tumor response to PARP inhibitors according to previous research studies [38].

Currently, PARP inhibitors are the best option for the maintenance treatment of advanced ovarian epithelial carcinoma. However, the intrinsic or acquired drug resistance of PARP inhibitors has gradually become an important clinical problem due to the wide use of these molecules [71]. Mutations in BRCA genes, such as deletions or insertions within pathogenic regions leading to the reopening of the gene open reading frame, as well as pathogenic site restoration mutations, may contribute to PARP inhibitor resistance [72]. To solve this problem, a potential combination of other medications is possible, such as olaparib and cediranib, which are used in patients with high-grade serous ovarian cancer who have experienced relapse or progression after PARP inhibitor maintenance therapy. However, the selection of the best drug combination to overlap PARP inhibitor resistance remains ambiguous.

For borderline ovarian tumors, surgery remains the primary treatment method, given that chemotherapy is not preferred due to its limited efficiency. Encouragingly, recent research has unveiled a potential new drug in the form of bractoppin, targeting the BRCA1-BRCT domains. This innovative medication aims to impede the growth of organoids by promoting apoptosis, inhibiting tumor growth, and triggering the expression of genes associated with DNA double-strand breaks (DSBs) [72]. Improving cancer prognosis could help optimize combination therapy on specific tumor molecular signaling pathways and achieve the synergistic effect of drugs [72].

Another alternative to conventional therapy is the use of nanotechnology, which is considered one of the upcoming cancer treatment approaches. Nanotechnology is based on the distinction between normal and malignant cells, according to the effect of nanocarriers action on both cell types. Some of the nanocarriers used are dendrimers, polymer micelles, polymer nanoparticles, and liposomes, which have different surface properties in term of surface chemistry, morphology, and mode of action. Nanoparticles ensure efficient drug delivery to alter the epigenetic modifications during epigenetic therapy. In siRNA-based nano-therapy, specialized target-modified nanoparticles are used to prevent the internalization problem. This technique enhances the anti-tumoral activity of siRNA by allowing unrestricted flow into the tumor vasculature and efficient intracellular transport. Nanoparticles can also be used in photodynamic therapy as depots for photosensitive drugs [73].

## 9. Discussion

Cancer is a major global public health problem with millions of new cases and deaths worldwide each year [74]. Significant progress has been made in elucidating the underlying molecular mechanisms involved in the detection and repair of DNA damage caused by ionizing radiation, environmental factors, and carcinogens. The importance of a DNA-damage monitoring network becomes clear when considering the links between the failure of the DNA damage-repair system, the development of congenital chromosomal instability syndromes and the onset of cancer [75]. In the present review, we describe the vital role of BRCA in the DNA damage repair network and its relationship with other related proteins, such as p53, BARD1, and BASC. The key role of the BRCA1 protein in DNA damage repair is mediated by its C-terminal domain, the BRCT domain. Understanding the cellular functions of BRCA1 helps identify new genes and proteins involved in cancer susceptibility and development [6]. We have carried out a detailed description of the BRCA-BRCT domain structure and its functions in various cellular signaling pathways related to tumor suppression as well as the importance of associated mutations in cancer development and susceptibility.

Reported mutations in BRCA1 are considered functionally deleterious [24]. Disease-associated amino acid changes in BRCT domains are categorized as unclassified in the Breast Cancer Information Care (BIC) [76]. This ambiguity in classification arises from the lack of allele testing in the general population or unclear allele segregation within families. Genetic testing for BRCA mutations has been proven crucial in identifying carriers susceptible to developing cancer [63]. Additionally, it facilitates the discovery of founder mutations within specific populations and determines the prevalence of BRCA mutations. This in turn allows the establishment of new medications and specific treatment strategies. The advancement of in silico techniques, including molecular docking and artificial intelligence, is playing a pivotal role in developing new treatments that are not only efficient but also tailored to the individual patient [77]. Understanding the structural bioinformatics analysis of BRCT is a critical step toward improving diagnosis and designing novel therapeutic agents.

## 10. Conclusions

Cancer is a major global health challenge, resulting in millions of deaths annually. Understanding in more detail the molecular mechanisms that govern DNA damage and repair is crucial, as the failure of DNA repair systems is the major cause of cancer initiation. This review emphasizes BRCA1’s pivotal role in the DNA damage repair network, highlighting its interaction with proteins, such as p53, BARD1, and BASC. BRCA1’s crucial function is mediated through its C-terminal BRCT domain. Understanding the diverse functions of BRCA-BRCT can help to identify cancer susceptibility genes and underscore the significance of associated mutations in cancer development. Many reported mutations in BRCA1-BRCT have been considered functionally deleterious. Genetic testing for BRCA-BRCT mutations is critical for identifying cancer susceptibility, discovering founder mutations and determining mutation prevalence. This can enable targeted medications and new treatment strategies. Moreover, structural bioinformatics analysis of BRCT is recognized as an important tool to further improve diagnosis and design novel therapeutic approaches. Overall, this exploration of BRCA’s role, mutations, and advances in genetic testing contributes to the ongoing efforts to combat cancer at both molecular and clinical levels.

## Figures and Tables

**Table 1:** Compilation of some well-characterized missense BRCA1-BRCT mutations and their possible deleterious effects on the structure and function of the BRCT protein.

Mutation	Gene Location	Variant Significance	Effect on BRCT	Reference
Lys1702 Thr	c.5105A>C	Unknown	Destabilize the interaction with Ser group of BACH1/CtIP proteins.	[28]
Arg1699Pro	c.5096G>C	Likely pathogenic and uncertain significance	Affect residues in the surface cleft of BRCT domain structure. Disrupt interaction with pSer990 in BACH1 protein: a 20-fold lower binding affinity.	[16]
Val 1696 Leu	c.5086G>C	Uncertain significance	Affect the interaction between BRCT domain BACH1 protein (50% reductions in BACH1 binding in rat).	[29]
Ala1708Glu Met1775Arg	c.5123C>A c.5324T>G	Pathogenic	Ablate the double-strand break repair and transcription function of BRCA1. Destabilize the BRCT fold. Inhibit BRCT interactions with histone deacetylases BACH1, and the transcriptional co-repressor CtIP.	[30]
Gly1763Val Leu1786Pro	5407 G>T 5476 T>C	Pathogenic Pathogenic	These mutations are far away from the interaction site between BRCA1 and its phosphor-ligands, which may at least partially explain why these two mutants have no deleterious effect on BRCA1 function.	[15]
Tyr 1853 Asn	c.5558A>G	Pathogenic/Likely pathogenic	Deleterious impact on protein structure and function. Impact phosphopeptide binding	[9]

**Table 2:** Current guidelines from the National Comprehensive Cancer Network (NCCN) for ovarian and breast cancer [63].

Criteria	Details
Young	Cancer diagnosis occurring at a younger age than usual. Breast cancer diagnosed in individuals under the age of 50.
Rare	Ovarian cancer, breast cancer in men, and pancreatic cancer among blood relatives.
Bilateral	Cancer affecting both organs in a pair, such as both breasts.
Multiple	Multiple instances of cancer or multiple primary cancers in a single individual. Same or related cancer occurring in two or more blood-related family members. Several generations being affected.
Familial mutation	An identified genetic mutation in a family member.
Ethnic predisposition	Jewish ancestry of Ashkenazi origin.
